# An Accurate Direction Finding Scheme Using Virtual Antenna Array via Smartphones

**DOI:** 10.3390/s16111811

**Published:** 2016-10-29

**Authors:** Xiaopu Wang, Yan Xiong, Wenchao Huang

**Affiliations:** School of Computer Science and Technology, University of Science and Technology of China, Hefei 230000, China; wangxp88@mail.ustc.edu.cn (X.W.); yxiong@ustc.edu.cn (Y.X.)

**Keywords:** indoor direction finding, antenna array simulation, multiple signal classification, multipath effect

## Abstract

With the development of localization technologies, researchers solve the indoor localization problems using diverse methods and equipment. Most localization techniques require either specialized devices or fingerprints, which are inconvenient for daily use. Therefore, we propose and implement an accurate, efficient and lightweight system for indoor direction finding using common smartphones and loudspeakers. Our method is derived from a key insight: By moving a smartphone in regular patterns, we can effectively emulate the sensitivity and functionality of a Uniform Antenna Array to estimate the angle of arrival of the target signal. Specifically, a user only needs to hold his smartphone still in front of him, and then rotate his body around $360^{\circ }$ duration with the smartphone at an approximate constant velocity. Then, our system can provide accurate directional guidance and lead the user to their destinations (normal loudspeakers we preset in the indoor environment transmitting high frequency acoustic signals) after a few measurements. Major challenges in implementing our system are not only imitating a virtual antenna array by ordinary smartphones but also overcoming the detection difficulties caused by the complex indoor environment. In addition, we leverage the gyroscope of the smartphone to reduce the impact of a user’s motion pattern change to the accuracy of our system. In order to get rid of the multipath effect, we leverage multiple signal classification to calculate the direction of the target signal, and then design and deploy our system in various indoor scenes. Extensive comparative experiments show that our system is reliable under various circumstances.

## 1. Introduction

Nowadays, localization technology has been widely applied in mobile social networking, augmented reality, etc. However, most of the applications are based on Global Positioning System (GPS). Due to the coarse-grained results of the GPS, these schemes cannot be used in the complex indoor environment. For instance, the GPS-based localization systems are not precise enough for users to locate a restroom in a shopping mall or an elevator in a hospital. Hence, an accurate indoor localization solution becomes an urgent need for many common users.

An indoor direction finding technique is the basis of the indoor localization technology. Many prior indoor direction finding systems detect the direction of signal source using antenna arrays. They steer the array’s beam to detect the direction of maximum energy using multiple signal classification. This direction corresponds to the signal’s spatial angle of arrival. Although these solutions are accurate and efficient, to gain the narrow beam and achieve a precise resolution, they need a large antenna array with a number of antenna elements that would result in a cumbersome and expensive device and impose restrictions on regular users in daily life.

We propose an accurate and lightweight indoor direction finding system that takes the advantages of an antenna array while avoiding its disadvantages. It utilizes the movement of the smartphone to emulate an antenna array. In brief, the smartphone samples the received signal at successive locations in space, as if we had a receive antenna at each of these points. By treating consecutive time samples as spatial samples, we can imitate an antenna array and leverage it to determine signal’s spatial angle of arrival.

Our research subjects are the high-frequency narrow-band acoustic signals. We set several loudspeakers emitting acoustic signals in the indoor environment. Then, we move the smartphone horizontally and keeping it steady in regular patterns with an approximate constant velocity for a period of time. During this sampling process, the acoustic samples are gathered by the microphone of the smartphone. After applying the noise reduction and signal optimizing, we imitate a virtual antenna array to process the received signals. Eventually, we leverage the multiple signal classification (MUSIC) algorithm [1] to estimate the direction of arrival of the target signals.

The Uniform Linear Arrays (ULA) are widely studied. We already did a lot of pre-studies about imitating a Virtual Uniform Linear Array (VULA) by moving the smartphone in front of the user’s body horizontally and steady to determine the direction of received signals [2]. However, the system based on the VULA is invalid under some particular circumstances. Through abundant experiments, we discover that the accuracy of the VULA system is limited not only by the azimuth angle of the target signal but also the elevation angle between the user and acoustic source. In practice, the smartphone and the loudspeaker are not always at the same height. In addition, during direction finding, when the user turns his/her back on the signal source will cause massive errors on the experiment results. We call it the blind-zone problem of the VULA system.

To the best of our knowledge, the following properties of Uniform Circular Arrays (UCA) make them attractive to our case. The UCA provides $360^{\circ }$ azimuthal coverage and also provides the information on source elevation angles. ULA, in contrast, provides only $180^{\circ }$ azimuthal coverage, and no results of the elevation angles. Moreover, the signal resolution processing by ULA is high at some particular arrival angles but low at others. However, the UCA has the same high signal resolution at around the $360^{\circ }$ azimuth. These advantages of UCA can solve the above problems.

**Figure 1:**
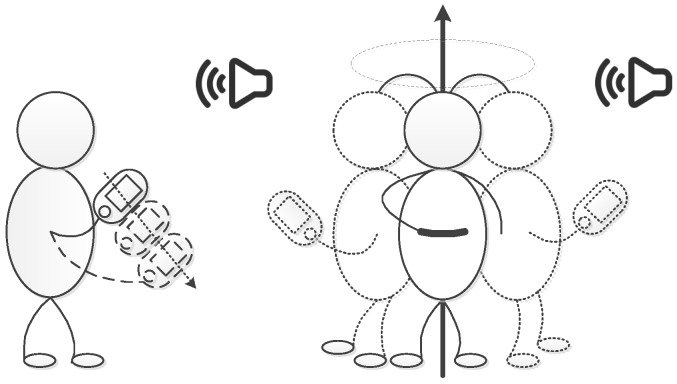
Virtual Uniform Linear Array and Virtual Uniform Circular Array system motion patterns of direction finding using a smartphone.

In Figure 1, for $360^{\circ }$ azimuthal coverage, we hold the smartphone still and rotate our body around $360^{\circ }$ at an approximate constant velocity during the sampling period to imitate a Virtual Uniform Circular Array (VUCA). However, in practice, the uses’ rotation velocity cannot sometimes be maintained approximately constant. Hence, we leverage the gyroscope of our smartphone to correct the sampled virtual nonuniform circular array to virtual uniform circular array under this particular circumstance. Then, we implement the MUSIC algorithm to calculate the direction of the target signals and solve the multipath effect problem.

We designed, deployed, and evaluated our system of direction finding in diverse circumstances. Our extensive experiment results show that our scheme supports high accuracy of direction finding. For phone to speaker direction finding, the mean error and the standard deviation of the measured angle are $3.4^{\circ }$ and $3.9^{\circ }$, respectively, within the range of 20 m.

The rest of the paper is organized as follows. Section 2 contains the work related to our research. Then, we present the preliminary approach in Section 3. After that, we introduce the design of acoustic signals direction finding system using VUCA in Section 4 and then report our experiment design and results in Section 5. Finally, we conclude the paper in Section 6.

## 2. Related Work

### 2.1. Direction Finding via Smartphones

As smartphones have become common personal belongings in daily life, more and more indoor direction finding systems are being deployed on smartphones. Swadloon [3] leverages the Doppler effects of the acoustic waves for direction finding and indoor localization. Users need to shake their smartphones and walk a few steps before using Swadloon. It only requires off-the-shelf speakers and supports an arbitrary number of users. However, this scheme does not take the multipath effect into consideration. In addition, it has a non-line of sight problem, which means if the user turns his back on the acoustic source, the results become unstable. Besides, there are other schemes solving the localization problems by Doppler effects, e.g., [4]. Zhang et al. [5] propose a way to locate outdoor access points (APs) using smartphones. Their insight is that, by rotating a wireless receiver (smartphone) around a signal-blocking obstacle (the user’s body), they can effectively emulate a directional antenna. By the periodic received signal strength (RSS) measurements of WiFi signals, the users can detect the outdoor APs’ locations. However, RSS measurements are easily affected by the multipath effect in indoor environments. It will cause a large angular error on the results of indoor direction finding. There are some similar methods using body as an antenna array for localization, e.g., [6]. In addition, there is an indoor localization technique based on an ambient sound fingerprint called the Acoustic Background Spectrum (ABS) [7]. As with other fingerprint-based localization techniques [8,9,10], location is determined by measuring the current fingerprint and then the ABS chooses the “closest" fingerprint from a database. This scheme is accurate and reliable, but it also demands a lot of preparation before localization, and it is easily affected by the environment change.

### 2.2. Localization Using Antenna Arrays

Antenna arrays are widely used in signals’ direction of arrival (DOA) estimation today. ArrayTrack [11] presents the design and experimental evaluation of an indoor localization system that uses MIMO-based (Multiple Input and Multiple Output) techniques to track wireless clients at a very fine granularity in real-time. The ArrayTrack AP system is built on two specialized devices called Rice WARP (Windows Advanced Rasterization Platform) FPGA (Field-Programmable Gate Array) based wireless radios. However, these specialized devices are inconvenient for daily use. Besides, there are some other related works using antenna arrays for localization, e.g., [12,13,14,15,16,17,18].

Adib et al. [19] use MIMO interference nulling to eliminate reflections off of static objects and focus the receiver on a moving target. Then, it tracks human beings by treating the motion of a human body as an antenna array and tracking the resulting RF (radio frequency) beam. This research utilizes a technique called inverse synthetic aperture radar (ISAR), which has been used for mapping the surfaces of the Earth and other planets. ISAR uses the movement of the target to emulate an antenna array. There are some other related works based on RF beam, e.g., [20,21,22].

### 2.3. Leveraging the Acoustic Wave by Smartphone

Leveraging the acoustic wave via smartphones for localization has been well addressed at present. Most of them leverage the low speed of the acoustic wave compared to wireless signals, such as the mechanism of TOA (time-of-arrival) and TDOA (time-difference-of-arrival) [23,24,25]. Yang et al. [26] address the fundamental problem of distinguishing between a driver and passenger using a mobile phone. This acoustic approach has the phone send a series of customized high frequency beeps via the car stereo. Beepbeep [27], which leverages COTS (Commercial off-the-shelf) Mobile Devices, is a pure software-based solution and uses only a speaker, a microphone, and some forms of device-to-device communication. It detects the distance between two smartphones with high accuracy. More importantly, this scheme is extremely lightweight. Inspired by the idea of relative positions of peer devices nearby as unique physical constraints on the possible location of a smartphone, Liu et al. [28] propose a peer assisted localization approach that leverages much more accurate distance estimate through acoustic ranging. Nandakumar et al. [29] raise an acoustic ranging based localization scheme. The basic idea of this scheme is to perform localization using acoustic ranging with respect to multiple landmarks in known locations. Furthermore, Qiu et al. [30] present a solution for achieving high speed 3D continuous localization for phone-to-phone scenarios. Their basic approach uses acoustic cues based on time-of-arrival and power level. Besides, there are other locaization technologies leveraging acoustic signals, e.g., [31,32,33].

### 2.4. Multiple Signal Classification (MUSIC)

Multiple Signal Classification (MUSIC) algorithm was proposed by Schmidt in 1979. MUSIC algorithm provides asymptotically unbiased estimates of

1. number of incident wavefronts present;
2. directions of arrival;
3. strengths and cross correlations among the incident waveforms;
4. noise/interference strength.

Due to the reliable results and anti-multipath characteristic of this approach, MUSIC algorithm is very general and can be applied widely in signal processing. Much advanced research of indoor localization is based on the MUSIC algorithm.

## 3. Basic Antenna Array Simulation

**Figure 2:**
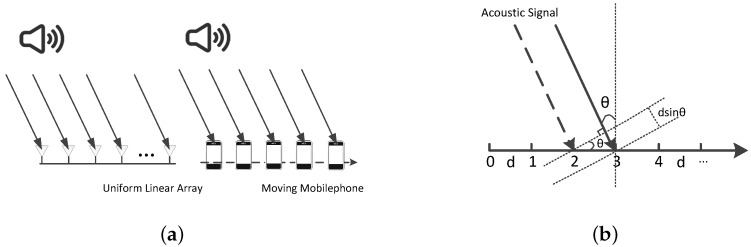
(**a**) Uniform linear array simulation; (**b**) virtual uniform linear arrays geometry analysis.

Figure 2 shows that an antenna array is able to locate the acoustic source by steering its beam spatially. Now, we treat these antenna elements as several smartphones that have the same model and process the target signal in the same way. Then, we can hold only one smartphone in our hand and move it through the same positions of these antenna array elements. The smartphone receives acoustic signals at successive locations at short distances. At each point in time, we capture a single measurement from the whole sampling data as if we had a receive antenna array at each of these sampling points. We assume that the moving velocity of the smartphone is approximatively constant. Thus, if we choose a fixed sampling interval, these points will be evenly distributed on the smartphone’s moving trajectory. Under the above assumption, we can leverage a normal smartphone to imitate an virtual uniform linear antenna array (VULA).

Then, we filter the signals with BPF (Band Pass Filter) to get rid of the interference of the noise. In addition, in order to facilitate the calculation, we leverage AGC (Automatic Gain Control) to eliminate variational amplitude of the target signal to a constant one. Eventually, we utilize a smoothed MUSIC algorithm to estimate the DOA of the target acoustic signal.

After deploying the VULA system in practice, some drawbacks are discovered. Due to the limit of the measuring range, VULA does not function well when the azimuth is over $180^{\circ }$. Moreover, the existence of elevation between the user and the acoustic source will also cause estimate errors of direction finding.

## 4. Design

### 4.1. Uniform Circular Array Simulation

Now, we solve the problems above by leveraging the VUCAs. We use the similar method of imitating the VULA system. The user holds his/her smartphone still and rotates around his/her body for $360^{\circ }$ at an approximate velocity. During the sampling period, we capture a single measurement in a particular sampling interval *T* as if we have a receive antenna array at each of these sampling points. These points are uniformly distributed on the moving circular trajectory.

**Figure 3:**
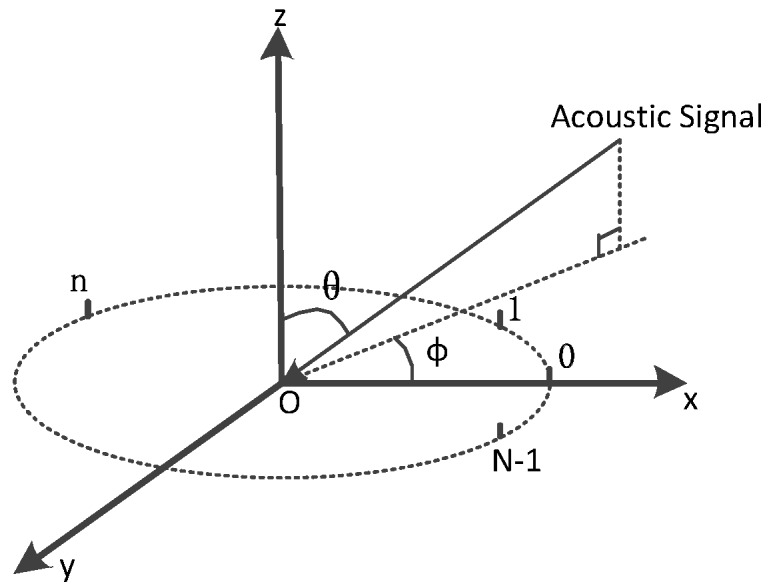
Virtual uniform circular arrays geometry analysis.

According to the VUCA geometry in Figure 3, we regard the first sample point during rotation as antenna element 0. Assuming the user rotates at the constant angular velocity *ω*, the antenna elements are uniformly distributed over the circumference of a circle of radius *r*. In our case, the radius stands for the distance between users’ hand and body (our default is *r* = 0.3 m for human beings of an average height). The *x*-axis lies on the line which connects the antenna element 0 with the circle center *O*. $\theta \in [0,\frac{\pi }{2}]$ is the source elevation angle that is measured down from the *z*-axis. The azimuth angle ϕ∈[0,2π] is measured from the *x*-axis. Since we choose the first sample point as the element 0 and the user starts rotating right in front of his/her body in most cases, the ground truth of our system is easy to detect.

We treat a single measurement as antenna element *n*, and there are *N* elements in total during sampling. If the *N* is large enough, the trajectory of the smartphone during the whole sampling period can be seen as a closed circle. Hence, the array element can be displaced by angle $\gamma_{n}$ from the *x*-axis:

(1) $$\gamma_{n}=n\omega T\approx \frac{2\pi n}{N};\;\;\;\;\;\;n=0,1,\cdots ,\left(N-1\right).$$

However, assuming the user rotates $360^{\circ }$ at a constant angular velocity *ω*, we can represent $\gamma_{n}$ using only *N* and *n* approximatively and refrain from the fact that we do not know the exact velocity of the smartphone. Then, the position vector of element *n* is:

(2) $$\vec{p_{n}}=\left(r\cos\gamma_{n},r\sin\gamma_{n},0\right).$$

The unit vector of the incoming acoustic signal, which is defined by elevation angle *θ* and azimuth angle ϕ, has this form:

(3) $$\hat{\gamma }=\left(\sin\theta \cos\phi ,\sin\theta \sin\phi ,\cos\theta \right).$$

For the purpose of calculating the phase differences between the signals received at the origin and at element *n*, we need to represent the time required for the signals to travel from these two points $\tau_{n}$:

(4) $$\tau_{n}=\frac{\hat{\gamma }\vec{p_{n}}}{c}=\frac{r}{c}\sin\theta \cos\left(\phi -\gamma \right).$$

The calculation of direction vector is similar with the Uniform Linear Arrays processing above. Let vector $\vec{\theta }=\left(\beta ,\phi \right)$ represent signal arrival direction. It can be expressed in the following equation:

(5) $$A\left(\vec{\theta }\right)=\left[\begin{array}{c} e^{j\beta \cos(\phi -\gamma_{0})}\\ e^{j\beta \cos(\phi -\gamma_{1})}\\ \cdots \\ e^{j\beta \cos(\phi -\gamma_{N-1})} \end{array}\right],$$

where *β* is a parameter to define the value of elevation angle *θ*, and *c* is the velocity of acoustic signals:

(6) $$\beta =\frac{2\pi f_{a}}{c}r\sin\theta .$$

Here, $f_{a}$ is the center frequency of the target acoustic signal.

### 4.2. Phase Mode Excitation of Continuous Circular Aperture

Unfortunately, the calculated direction vector is not a Vandermonde matrix. In order to leverage MUSIC algorithm to determine the direction of acoustic source, we need to construct a preprocessing matrix for $A(\vec{\theta })$ via the phase mode excitation technique.

We consider the case of a continuous circular aperture. The excitation functions are periodic with period 2π and can be represented in terms of a Fourier series. An arbitrary excitation function w(γ) is determined by following formula:

(7) $$w\left(\gamma \right)=\sum_{m=-\infty }^{+\infty }c_{m}e^{jm\gamma },$$

where the *m*th phase mode $w_{m}\left(\gamma \right)=e^{jm\gamma }$ is a spatial harmonic of the array excitation and $c_{m}$ is the corresponding Fourier series coefficient. As the result of aperture exciting with the *m*th phase mode, the normalized far-field pattern is:

(8) $$f_{m}^{c}\left(\vec{\theta }\right)=\frac{1}{2\pi }\int_{0}^{2\pi }w_{m}\left(\gamma \right)e^{j\beta \cos(\phi -\gamma )}\;d\gamma$$

where the superscript *c* represents the continuous aperture. According to $w_{m}\left(\gamma \right)$, the far-field pattern can be expressed as:

(9) $$f_{m}^{c}\left(\vec{\theta }\right)=j^{m}J_{m}\left(\beta \right)e^{jm\phi },$$

where $J_{m}\left(\beta \right)$ is the Bessel function of the first kind of order *m*. It contains the information of the elevation angle and the amplitude. Due to the Automatic Gain Control (AGC) we utilize, the amplitude of the acoustic signal is replaced by another one that is close to constant. In addition, we can easily detect that the far-field signal pattern and the excitation function share the same azimuthal angle change $e^{jm\phi }$.

However, the number of modes that can be excited is limited. Now, we let *M* denote the highest order mode that can be excited by the aperture at a reasonable strength (m<M). We can say that visible region $\theta \in [\frac{\pi }{2}]$ translates into $\beta =\frac{2\pi f_{a}}{c}r\sin\theta \in \left[0,\frac{2\pi f_{a}r}{c}\right]$. The mode amplitude $J_{m}\left(\beta \right)$ is small when the Bessel function order *m* exceeds its argument *β*. If m≥M, $f_{m}^{c}\left(\vec{\theta }\right)$ will be small over the entire visible region. Thus,

(10) $$M\approx \left\lfloor \frac{2\pi f_{a}}{c}r\right\rfloor .$$

### 4.3. Phase Mode Excitation of Uniform Circular Array

Now, we consider phase mode excitation of an *N* elements virtual uniform circular array (VUCA) in our case. The normalized beamforming weight vector that excites the array can be expressed as:

(11) $$w_{m}^{H}=\frac{1}{N}\left[e^{jm\gamma_{0}},\cdots ,e^{jm\gamma_{N-1}}\right].$$

Then, we can get the resulting array pattern $f_{m}^{s}\left(\theta \right)$:

(12) $$f_{m}^{s}\left(\vec{\theta }\right)=w_{m}^{H}A\left(\vec{\theta }\right)=\frac{1}{N}\sum_{n=0}^{N-1}e^{jm\gamma_{n}}e^{j\beta \cos(\phi -\gamma_{n})},$$

where the superscript *s* denotes the sampled aperture is discrete. As a result of |m|<N,

(13) $$\begin{array}{cc} f_{m}^{s}\left(\vec{\theta }\right)= & j^{m}J_{m}\left(\beta \right)e^{jm\phi }\\ & +\sum_{q=1}^{+\infty }\left[{\left(j\right)}^{g}J_{g}\left(\beta \right)e^{ig\phi }+{\left(j\right)}^{h}J_{h}\left(\beta \right)e^{jh\phi }\right]. \end{array}$$

In the above equation, g=Nq-m and h=Nq+m. The first term in this equation, the principal term, is the same as the far-field pattern of Equation (9) corresponding to the continuous aperture case. The remaining terms are caused by the sampling of the continuous aperture, and they are the residual terms to our case. After setting the perspective from Equation (13), we come to the conclusion that the residual terms can be ignored if N>2M. Due to the attribute of $J_{-m}\left(\beta \right)={\left(-1\right)}^{m}J_{m}\left(\beta \right)$, the UCA array pattern for mode *m* is:

(14) $$f_{m}^{s}\left(\vec{\theta }\right)\approx {\left(j\right)}^{\left|m\right|}J_{\left|m\right|}\left(\beta \right)e^{jm\phi }.$$

### 4.4. Beamforming Matrix and Direction Vector Construction

With this background on phase mode excitation of circular arrays, we introduce UCA-MUSIC algorithm [34] to solve the direction finding problem. For the purpose of making the transformation of direction vector $A(\vec{\theta })$ from element space to beamspace, we need to introduce the beamformer $F_{r}^{H}$:

(15) $$B\left(\vec{\theta }\right)=F_{r}^{H}\cdot A\left(\vec{\theta }\right).$$

In order to simply the calculation, we also present $F_{e}^{H}$, which is a intermediate quantity.

(16) $$F_{e}^{H}=C_{v}V^{H},$$

where

(17) $$C_{v}=diag\left\{j^{-M},\cdots ,j^{-1},j^{0},j^{-1},\cdots ,j^{-M}\right\},$$

and

(18) $$V=\sqrt{N}\left[w_{-M},\cdots ,w_{M}\right].$$

Equation (17) helps to eliminate the term ${\left(j\right)}^{\left|m\right|}$ in Equation (14). In Equation (11), the column vector $w_{m}^{H}$ has been defined. According to the above discussion, we can get:

(19) $$F_{e}^{H}A\left(\vec{\theta }\right)=C_{v}V^{H}A\left(\vec{\theta }\right)=\sqrt{N}J\left(\beta \right)v\left(\phi \right).$$

The information of the azimuthal angle ϕ is only in the vector v(ϕ):

(20) $$v\left(\phi \right)={\left[\begin{array}{ccccccc} e^{-jM\phi }, & \cdots , & e^{-j\phi }, & 1, & e^{j\phi }, & \cdots , & e^{jM\phi } \end{array}\right]}^{H}.$$

Through observation, this is similar in Vandermonde form to the VULA direction vector. J(β) contains the information of elevation angle *θ*:

(21) $$\begin{array}{c} \begin{array}{cccccccc} J(\beta )=diag[J_{M}\left(\beta \right), & J_{M-1}\left(\beta \right), & \cdots , & J_{1}\left(\beta \right), & J_{0}\left(\beta \right), & J_{1}\left(\beta \right), & \cdots , & J_{M}\left(\beta \right)] \end{array} \end{array}.$$

Now, we introduce $W^{H}$ that has centro-Hermitian rows. The $F_{r}^{H}$ is constructed by premultiplying $F_{e}^{H}$ by $W^{H}$. The beamformer $F_{r}^{H}$ can be expressed like this:

(22) $$F_{r}^{H}=W^{H}F_{e}^{H}=W^{H}C_{v}V^{H}.$$

Thus, the beamspace manifold vector $B(\vec{\theta })$ will be:

(23) $$B\left(\vec{\theta }\right)=F_{r}^{H}A\left(\vec{\theta }\right)=\sqrt{N}W^{H}J\left(\beta \right)v\left(\phi \right).$$

The multiplication of two centro-Hermitian vectors can construct a real-valued beamspace manifold vector. Thus, *W* must satisfy $\tilde{I}W=W^{*}$:

(24) $$W=\frac{1}{\sqrt{M^{\prime }}}\left[\begin{array}{ccccc} v(\alpha_{-M}), & \cdots , & v(\alpha_{0}), & \cdots , & v(\alpha_{M}) \end{array}\right],$$

where $M^{\prime }$ represents the total number of *M*. $M^{\prime }=2M+1$ and $\alpha_{i}=\frac{2\pi i}{M^{\prime }}\left(i\in \left[-M,M\right]\right)$.

Through observation, we learn that, during the progress of construction, a real-valued beamspace manifold vector is also actually the progress of smoothing. It helps the algorithm to eliminate the adverse impact of coherent signal in the environment.

### 4.5. Estimating Direction of Arrival by the MUSIC Algorithm

The smartphone receives the acoustic signals at sampling point *n*: $x\left[t_{n}\right]=A\left(\vec{\theta }\right)S\left(t_{n}\right)+\sigma \left(t_{n}\right)$ [35]. Due to the BPF we leverage, the noise $\sigma (t_{n})$ is eliminated. The UCA-RB-MUSIC algorithm utilizes the beamformer $F_{r}^{H}$ to make the transformation from element space to beamspace:

(25) $$y\left[t_{n}\right]=F_{r}^{H}x\left[t_{n}\right]=B\left(\vec{\theta }\right)S\left(t_{n}\right).$$

Thus, the corresponding beamspace covariance matrix is:

(26) $$R_{y}=E\left[y\left[t_{n}\right]y{\left[t_{n}\right]}^{H}\right].$$

The next step is the eigenvalue decomposition of covariance matrix $R=Re\{R_{y}\}$, and we introduce the signal-subspace and noise-subspace. We use $U_{n}=\left(v_{2},\cdots ,v_{M^{\prime }}\right)$ to represent noise eigenvectors of noise-subspace listed in descending order.

Eventually, the MUSIC spectrum has this form:

(27) $$P\left(\vec{\theta }\right)=\frac{1}{B^{T}\left(\vec{\theta }\right)U_{n}U_{n}^{T}B\left(\vec{\theta }\right)}\propto \frac{1}{v^{H}\left(\phi \right)J\left(\beta \right)\left(WU_{n}U_{n}^{T}W^{H}\right)J\left(\beta \right)v\left(\phi \right)}.$$

We can estimate that the direction of the target signal depends on the 2D search for peaks in the spectrum $P\left(\vec{\theta }\right)=P\left(\beta ,\phi \right)$. The elevation angle affects the spectrum through the parameter $\beta =\frac{2\pi f_{a}}{c}r\sin\theta$. The azimuth angle ϕ(∈0,2π) signifies that our system of indoor direction finding is omni-directional.

### 4.6. Search for Peaks Using Fast Fourier Transformation

For the purpose of calculating the 2D search for peaks in the spectrum, we leverage the Fast Fourier Transformation (FFT) to calculate the azimuth angle on a set of given elevation angles.

According to Equation (27), we let

(28) $$V\left(\phi ;\beta \right)=v^{H}\left(\phi \right)J\left(\beta \right)\left(WU_{n}U_{n}^{T}W^{H}\right)J\left(\beta \right)v\left(\phi \right)$$

donate the null spectrum at the elevation specified by *β*. We also let $Q_{\beta }=J\left(\beta \right)\left(WU_{n}U_{n}^{T}W^{H}\right)J\left(\beta \right)$, and then we can get

(29) $$V\left(\phi ;\beta \right)=\sum_{l=-(M^{\prime }-1)}^{M^{\prime }-1}a_{\beta }\left(l\right)e^{jl\phi },$$

where $a_{\beta }\left(l\right)=\sum_{i,j:j-i=l}Q_{\beta }\left(i,j\right)$. The matrix $Q_{\beta }$ is Hermitian, so $a_{\beta }\left(-l\right)=a_{\beta }^{*}\left(l\right)$. Now, we can express the null spectrum in terms of the discrete time Fourier transform of the $M^{\prime }$ point sequence:

(30) $$a_{\beta }^{\prime }=\left\{a_{\beta }\left(0\right),2a_{\beta }\left(-1\right),\cdots ,2a_{\beta }\left(-M^{\prime }+1\right)\right\}.$$

Finally, we get

(31) $$V\left(\phi ;\beta \right)=Re\{A_{\beta }^{\prime }\left(\phi \right)\},$$

where $A_{\beta }^{\prime }=\sum_{l=0}^{M^{\prime }-1}=a_{\beta }^{\prime }e^{-jl\phi }$.

For a given elevation angle, the null spectrum can be evaluated at *L* equi-spaced azimuth angles $\phi_{l}=\frac{2\pi l}{L},L=0,1,\cdots ,L-1$ using an *L* points FFT of the sequence $a_{\beta }^{\prime }$, and when $L>M^{\prime }$, $a_{\beta }^{\prime }$ is appropriately zero padded.

### 4.7. The VULA and VUCA Algorithm Comparison

#### 4.7.1. The Measuring Range of Direction Finding

According to the direction vector of VULA: $A\left(\theta \right)={\left[\begin{array}{ccccc} 1 & e^{jQ} & e^{j2Q} & \cdots & e^{(N-1)jQ} \end{array}\right]}^{H}$ ($Q=\;2\pi f_{a}\frac{vTsin\theta }{c}$), we learn that when the azimuth angle $\theta_{1}=2\pi -\theta_{2}\left(\theta_{1}\neq \theta_{2}\right)$, $Q\left(\theta_{1}\right)=Q\left(\theta_{2}\right)$. This will cause the fuzzy phenomenon of the VULA system. In other words, the measuring range of VULA is $180^{\circ }$. On the other hand, each of the VUCA direction vector in Equation (23) is unique. Thus, the measuring range of VUCA is $360^{\circ }$.

#### 4.7.2. Signal Resolution

The resolution of received target signal source on a certain azimuth angle is related to the change rate of direction vector around this azimuth angle when leveraging the antenna array to detect the direction of arrival. The resolution and the change rate are positively related. Thus, we can measure the resolution by:

(32) $$D\left(\theta \right)=||\frac{dA(\theta )}{d\theta }|{|}_{F}.$$

Thus, the resolution of VULA system is

(33) $$D_{VULA}\left(\theta \right)=\sqrt{\sum_{i=-N}^{N}i^{2}}\frac{2\pi fvT}{c}\left|cos\theta \right|\propto |cos\theta |.$$

According to Equation (33), the resolution of the VULA system is changing in the form of cosine. It means when the signals source’s azimuth angle is perpendicular to the moving trajectory of moving smartphone, the resolution reaches the highest point. However, the resolution becomes lower and lower until the direction of the target signal becomes parallel to the moving trajectory.

On the other hand, the resolution of VUCA system is

(34) $$D_{VUCA}\left(\theta \right)=\sqrt{\sum_{i=-N}^{N}i^{2}}=k,$$

where *k* is a constant. It represents the VUCA system that has the same resolution of the received target signal source around $360^{\circ }$, which makes the VUCA system more stable than the VULA system during daily use.

### 4.8. Leveraging Gyroscope of the Smartphone

Our system is based on the hypothesis that users hold the smartphone still and rotate their body for $360^{\circ }$ in a constant speed. However, in practice, the moving velocity of the smartphone is always changing, which causes the VUCA to become a nonuniform circular array. Hence, we need to reduce the impact of changing velocity of the smartphone to our system.

**Figure 4:**
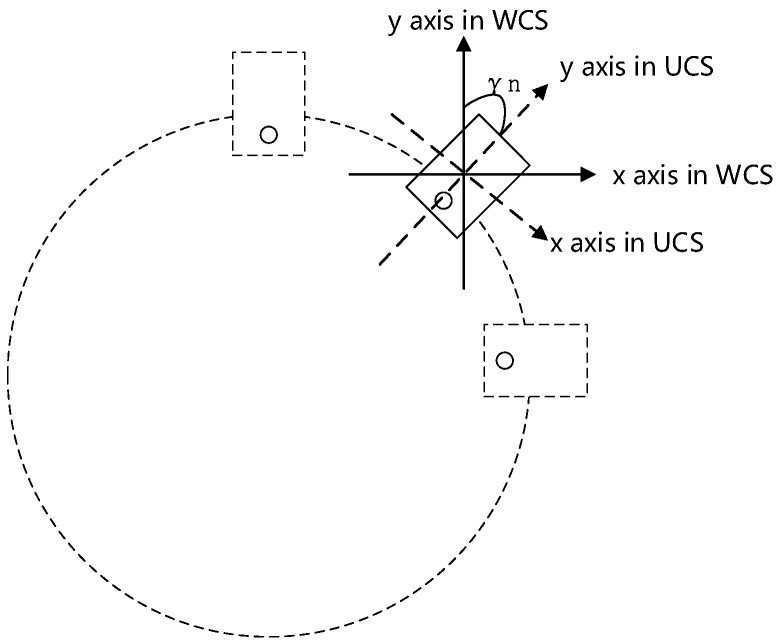
User’s phone coordinate system (UCS) to world coordinate system (WCS) when the phone is horizontal.

In order to reduce the impact of the inconstant velocity of body rotation, we need to calculate the rotated angle in each of the sampling points during the sampling process. For correcting the error caused by the nonuniform array, we decide to leverage the gyroscope of our smartphones to select the proper sampling point to construct the virtual uniform circular array. The gyroscopes are the common inner sensors of most smartphones. The gyroscope is available to measure the rotational velocities of the smartphone itself in three-dimensions. This means that we can detect the rotated angle of the moving smartphone in real-time by leveraging the gyroscope of the smartphone. In Figure 4, we assume that a user holds the smartphone still and rotates his body for $360^{\circ }$ at an arbitrary velocity. During the sampling period, the smartphone moves horizontally. According to the outputs of the gyroscope, we calculate the opening angle from the *y*-axis in the user’s phone coordinate system (UCS) to the one in a world coordinate system (WCS) by using the transform function from UCS to WCS. Hence, we let the sampling points uniformly distribute on the moving trajectory through setting a constant angle interval. In other words, we partition the samples by the constant angle interval instead of the time interval. Now, we represent the array elements by $\gamma_{n}$:

(35) $$\gamma_{n}=n\gamma \left(n=1,2,3...m;\gamma_{n}\leq 360^{\circ }\right).$$

Here, *m* is the number of sampling points and *γ* is the sampling interval that we set. Notice that the $\gamma_{n}$ here represents the position of VUCA’s array element, and it is independent of the azimuth angles of the target signals. However, the leveraging gyroscope of the smartphone during every sampling process will increase the calculation amount of our system. For optimizing system efficiency, we only leverage the VUCA-gyroscope system for direction finding under some particular circumstances. First, during the whole sampling period, we calculate the standard deviation of the shifty angular velocity of the body rotation by the outputs of the gyroscope. Then, according to the extensive experiments, we figure out the threshold of the standard deviation to distinguish whether the user is moving the smartphone at an approximate constant velocity or variable velocity. Finally, we use the VUCA-gyroscope system when the standard deviation of the angular velocity exceeds the threshold value. Otherwise, we use the common VUCA system. We will describe how to set the threshold in Section 5.6.

## 5. Performance Evaluation

Our scheme is deployed on a Samsung Note2 (Seoul, Korea) with an Android system. Several ordinary digital loudspeakers are chosen to be the acoustic sources. We set the speakers in various indoor environments as anchor points. The acoustic signals’ frequencies in the experiments are selected to be 19,500 Hz to 21,500 Hz. In practice, we can set the acoustic frequency to be higher than 20,000 Hz. This kind of signal cannot be heard by human beings, which has the minimal impact to users. High frequency acoustic signals are relatively seldom in the daily life unlike WiFi signals or light signals. The high frequency acoustic signals are less likely to be affected by other signals in the environment.

### 5.1. Experiment Design

**Figure 5:**
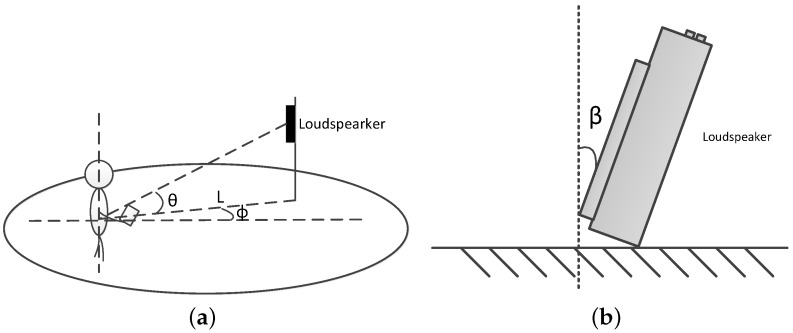
Experiment design. (**a**) the sketch of experiment design; and (**b**) the orientation angle *β* of the acoustic source.

The sketch of experiment design is shown in Figure 5a. The distance between the smartphone and the acoustic source is *L*. ϕ is the azimuth angle, *θ* is the elevation angle of the phone and the loudspeaker and the orientation angle of the acoustic source is *β*, which is shown in Figure 5b.

After we deploy the common loudspeakers in the indoor environment, we hold the smartphone right in front of our body and start rotating our body clockwise for $360^{\circ }$ with our system on the smartphone running. After the sampling process, we can read the azimuth angle and the elevation angle of the target speaker on the smartphone’s screen.

The main method of evaluating performance of our scheme is to vary *L*, ϕ, *θ* and *β* by deploying the loudspeakers and smartphones at different positions. The angular velocity of the body rotation has no particular restrictions. However, in most experiments, the experimenter rotates his body naturally and stable, during which the moving velocity of the smartphone can be treated as an approximately constant one. According to the gyroscope outputs in the experiments, the mean angular velocity of the smartphone is around 1.5 to 2.5 rad/s. We conduct the extensive comparison experiments to verify the influence of inconstant angular velocity to the accuracy of our system. We also conduct a lot of contrast experiments to the VULA direction finding and the VUCA direction finding in the same environment to verify the superiority of VUCA system. We measure ϕ and *θ* 50 times for each circumstance with both VULA system and VUCA system.

**Figure 6:**
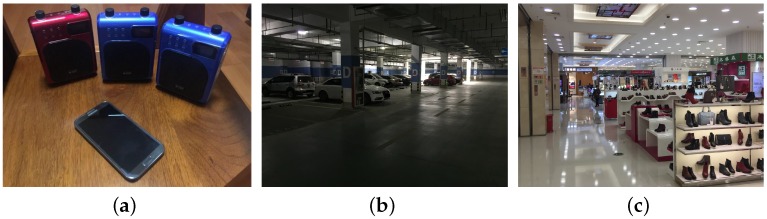
(**a**) experiment devices; (**b**) the underground parking lot; (**c**) the shopping mall.

Most of our experiments are conducted in an underground parking lot of a residential community. The devices that we use in the experiments are shown in Figure 6a. The real experiment scenario is shown in Figure 6b. There are several concrete pillars holding the ceiling with 0.8 m across. We conduct our experiments and vary the variables in the experiments as follows:

1. **Distance** *L***:** We fix the speakers on the pillars and leverage a laser range finder to detect the relative distance between the experimenter and loudspeakers;
2. **Azimuth angle** ϕ**:** We leverage a marker rod, a laser range finder and a protractor to construct a right-angled triangle on the ground. The vertexes of the triangle are the loudspeaker, the experimenter and the marker rod. The hypotenuse is *L*. Hence, we can vary different azimuth angles ϕ by adjusting the sides of the triangle;
3. **Elevation angle** *θ***:** As long as we know the distance *L*, we can set the speakers on different heights of the pillars to vary different elevation angles *θ*;
4. **Orientation angle of the loudspeaker** *β***:** We put the loudspeakers on the chairs or the cars. We try to keep the loudspeakers upright or lay them down while $\beta =0^{\circ }$ or $90^{\circ }$. We also place the loudspeakers while $\beta =45^{\circ }$ using a protractor and a support.

**Figure 7:**
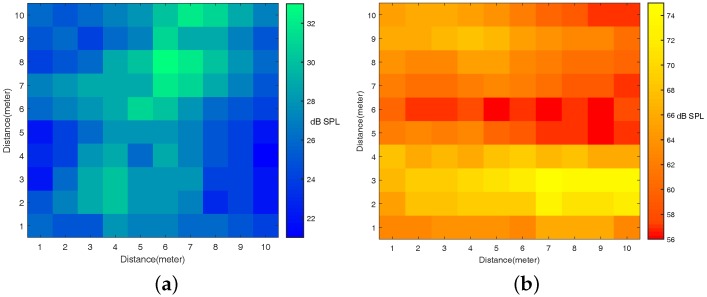
(**a**) the noise map of the underground parking lot; and (**b**) the noise map of the shopping mall.

In addition, we measure the noise maps of our experiment scenarios by the smartphone (underground parking lot and shopping mall, 10×10 m^2^) when there are no experimental loudspeakers in the environment. Figure 7a shows that the sound pressure of the underground parking lot is stably around 28 dB SPL (sound pressure level).

### 5.2. Indoor Environments with Single High Frequency Acoustic Waves

First, we deploy only one acoustic source in the indoor environment. Then, we obtain the anchor point’s direction by horizontally and steadily moving the phone in front of our body (VULA) or holding the phone still and rotating our body for $360^{\circ }$ (VUCA), and reading the results of direction finding from the phone.

#### 5.2.1. Affected by the Distance

The direction finding accuracies of different distances *L* are significant evaluations in our system. Hence, we set *L* = 5 m, 10 m, 20 m, 30 m, $\phi =30^{\circ },90^{\circ },240^{\circ }$, $\beta =0^{\circ }$, and the loudspeaker and the smartphone are at the same height, which means $\theta =0^{\circ }$.

**Figure 8:**
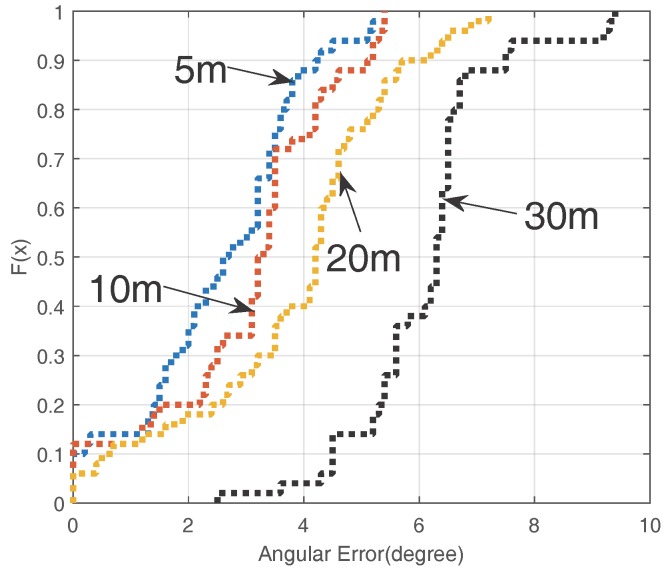
Cumulative distribution function (CDF) of the angular errors when using VUCA and *L* = 5 m, 10 m, 20 m, 30 m.

The cumulative distribution function (CDF) of the angular errors of the VUCA system is shown in Figure 8. We notice that the mean error of the VUCA system is less than $4^{\circ }$ when *L* < 20 m, and it is still acceptable when *L* = 30 m.

#### 5.2.2. Affected by the Azimuth Angle

**Figure 9:**
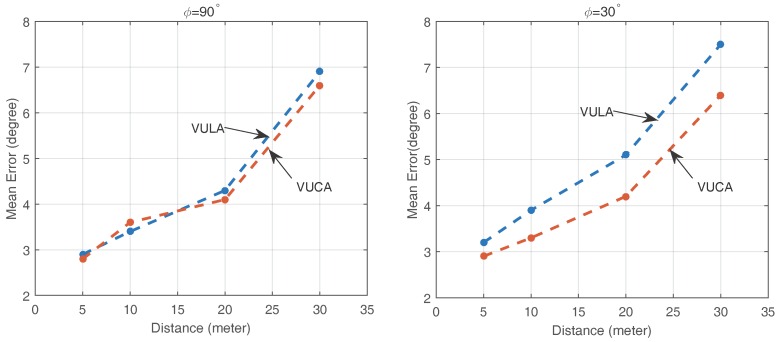
Mean error of direction finding when $\phi =90^{\circ }$ or $30^{\circ }$ with different distances *L*.

We plot the mean angular errors on different distances *L* = 5 m, 10 m, 20 m, 30 m, $\beta =\theta =0^{\circ }$ and azimuths $\phi =30^{\circ },90^{\circ },240^{\circ }$ between the smartphone and the speaker using both VULA and VUCA in Figure 9. Then, through the observation, there are not many differences between the direction finding performance of the VULA system and the VUCA system at the same distance between the acoustic source and the smartphone when $\phi =90^{\circ }$. However, when $\phi =30^{\circ }$, the VUCA obviously performs better than VULA.

We believe the reasons for this phenomenon are the fact that the VULA system’s resolution to the acoustic signal is changing, and it is related to the rate of change of the direction vector. The resolution reaches the highest level when the directions of the arrived signal are perpendicular to the axial line. When the signal comes close to the same direction along the axial line of VULA, the resolution gets lower and lower. However, the VUCA system’s resolution remains the same for $360^{\circ }$ duration.

Eventually, when $\phi =240^{\circ }$, the VULA is extremely unstable. In practice, we can not make sure that the acoustic source is right in front of us. Thus, we cannot afford the possible serious errors of the VULA system. This is why we choose to use the VUCA system.

#### 5.2.3. Affected by the Elevation

**Figure 10:**
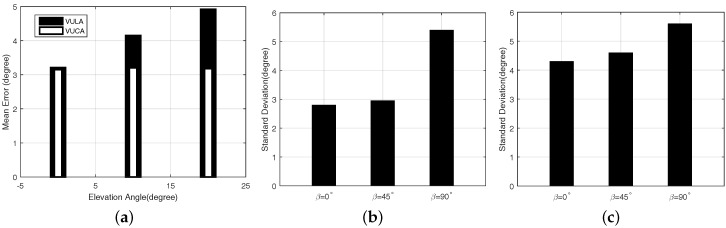
(**a**) mean error of VULA and VUCA when $\theta =0^{\circ };10^{\circ };20^{\circ }$ and *L* = 8 m; (**b**) standard deviations of angular error when L≤ 6 m; and (**c**) standard deviations of angular error when *L* = 24 m.

In this section, we test the angular errors when the speaker and the smartphone are not at the same height. We set *L* = 8 m, $\phi =45^{\circ }$ and $\theta =0^{\circ };10^{\circ };20^{\circ }$ and $\beta =0^{\circ }$. To vary different elevation angles, we fix the speakers on different heights of the pillars in the parking lot. We can find the different performances of the VULA system and the VUCA system in different scenarios in Figure 10a. We detect that the VULA is affected when the elevation angle *θ* grows. On the other hand, the VUCA’s accuracy remains almost the same as different elevation angles. We believe the main reason for these results is that the signal model of the VULA system is two-dimensional, while the signal model of the VUCA system is three-dimensional. In other words, only the VUCA system takes the elevation angle of the target signals *θ* into consideration. In addition, the VUCA system has another advantage that it can also calculate the specific value of elevation angle *θ* by the UCA-RB-MUSIC algorithm. For example, it helps us to determine whether the anchor points are on the same floor with us or not in a big shopping plaza. This characteristic can bring more productive experiences to the user.

#### 5.2.4. The Orientation Angle of the Acoustic Source

The loudspeaker we used for the experiment is the common speaker that can be found in daily life. In the experiments, we find that the different orientation angle *β* of the acoustic source will also affect the results.

Now, we evaluate the VULA and the VUCA system with different *β*. Figure 10b,c show the standard deviations of angular errors when $\beta =0^{\circ },45^{\circ },90^{\circ }$, L≤ 6 m, *L* = 24 m, $\theta =\;0^{\circ }$ and $\phi =\;45^{\circ }$. From these figures, we learn that the closer we get to the acoustic source, the easier direction finding accuracy is affected by the orientation angle *β*. We believe the reasons for this phenomenon are that the acoustic source we choose is not omni-directional, and when the user is close to the acoustic source, the signals reflected from the wall sometimes are stronger than those that directly go to the smartphone. Hence, the way of placing the loudspeakers also affects our results in some ways. However, when *L* = 24 m, the signals reflected from the wall become much weaker than those directly from the acoustic source. Thus, the orientation angle has less influence on our results.

#### 5.2.5. Multipath Effect

**Figure 11:**
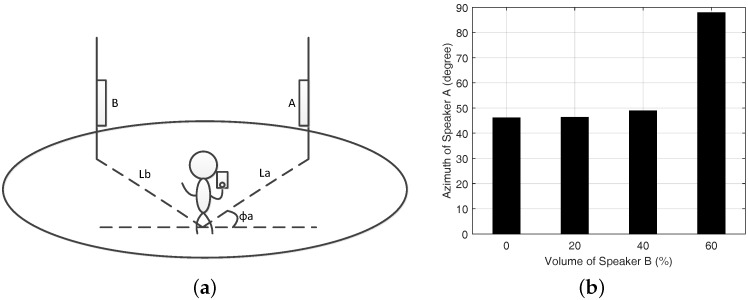
(**a**) multipath effect experiment; and (**b**) the mean azimuth of speaker A with different volumes of speaker B.

As the multipath effect has the most impact on the accuracy of indoor direction finding, we also test the VUCA system stability in the multipath signals environment. In brief, we set two loudspeakers A and B broadcasting the same frequency acoustic signals in an empty room. We also set $L_{A}=L_{B}=8m$, $\phi_{A}=45^{\circ }$, $\phi_{B}=135^{\circ }$ and $\theta =\beta =0^{\circ }$ in Figure 11a.

The volume of Speaker A is constantly 60%. Then, we change the volume of speaker B from 0% to 60% to emulate the impact of multipath effect on our results. The mean direction finding results are plotted in Figure 11b. When the volume of speaker B is set from 0% to 40%, it has little effect on our results due to the anti-multipath MUSIC algorithm.

We then conduct experiments with the receiver standing close to the wall and only one speaker broadcasting in the environment. We set $\phi =45^{\circ }$, θ=β=0 and *L* = 2, 8, 15, and 20 m. The standard deviation of the direction finding results is shown in Table 1.

From Table 1, we find that if the user stands close to a wall and a signal source at the same time, the VUCA system will become unstable. We believe that the reason is the signal reflected from the wall sometimes is stronger than the one directly from the acoustic source in this particular situation. Although such a circumstance is rare in practice, we still need to solve it in future work.

#### 5.2.6. Non-Line of Sight

We set and test a simple case on the effect by non-line of sight (NLOS). We let a person stand between the phone and acoustic source as a blocker (*L* = 10 m, $\beta =0^{\circ },\phi =45^{\circ }$ and $\theta =0^{\circ }$), and then we measure the standard deviation of the angular errors related to the different distances (1 m, 3 m, 5 m, 7 m, 9 m) from the blocker and the phone. In Table 2, we learn that when the person stands very close to the loudspeaker or the smartphone, the standard deviation slightly increases. We can say that the blocker between the acoustic source and the smartphone has very little impact on our experiment. In practice, it is very common to see a person passing by the line between our anchor points and the smartphone. Our system is still reliable in this circumstance. In addition, it is verified in the experiment with noisy environments.

Another case of NLOS is that the user turns his back on the source. We already explained why VULA can not detect the acoustic source behind the users. Our method of rotating the phone around the user’s body gets rid of this problem perfectly.

### 5.3. Indoor Environment with Multiple High Frequency Acoustic Waves

Our system is built on the assumption that a person in a library or a hospital can leverage their smartphone to locate the loudspeakers in the environment. However, in daily life, these speakers are used for other purposes most of the time, i.e., broadcasting music or announcements. Thus, we need to detect the robustness of our system when the the same acoustic source transmits multiple frequency acoustic signals.

**Figure 12:**
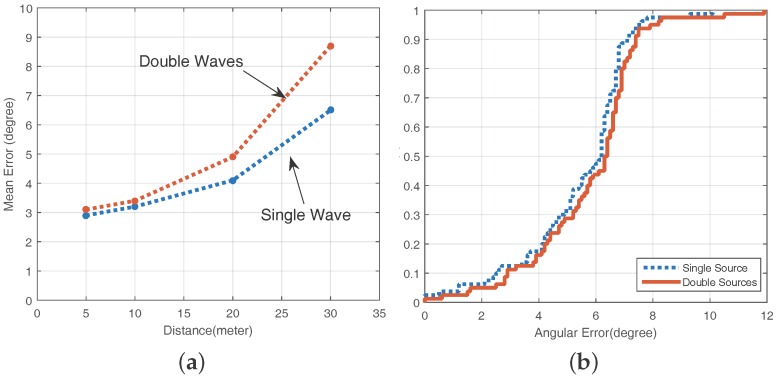
(**a**) the same acoustic source with single and multiple waves; and (**b**) the CDF of the angular error in a noisy environment.

We put only one speaker in the underground parking lot environment and then let the speaker broadcast a high frequency acoustic signal along with or without the common music. We also set *L* = 5 m, 10 m, 20 m, 30 m, $\beta =0^{\circ },\phi =45^{\circ }$ and $\theta =10^{\circ }$. In Figure 12a, we can detect that when L≤ 20 m, the VUCA system is stable for the acoustic source transmitting both single and multiple acoustic signals. When *L* > 20 m, the results have been affected.

The reason for the above-mentioned results is that when the speaker sends multiple signals, the signal strength of each component becomes weaker. The solution to this problem is that we can use the acoustic source with higher power. Fortunately, in the indoor application scenario such as a shopping mall or a hospital, there are high power speakers in a passageway or a conference room in most cases.

We also have another assumption that the different loudspeakers transmit various high frequency acoustic signals to distinguish different locations in indoor environments. If the user knows a specific frequency of his/her target acoustic signal, our system can guide the user to his/her destination. Thus, we also need to verify the direction finding ability of the VUCA system when the multiple acoustic sources transmit different high frequency acoustic signals in the environment.

Three loudspeakers are planted in the underground parking lot. These speakers broadcast acoustic signals with different frequencies of 19,500 Hz, 20,500 Hz and 21,500 Hz. In addition, the distances from all three loudspeakers to the user are the same. We treat one of the speakers as target speakers and let the relevant position of the user and the target speaker be *L* = 12 m, $\beta =0^{\circ },\phi =45^{\circ }$ and $\theta =0^{\circ }$. We evaluate the mean angular error of direction finding under single source, double source and triple source circumstances. From Table 3, we learn that the VUCA system functions well on the above assumption, and it is very crucial in daily use.

### 5.4. Noisy Environment

We deploy our VUCA system in a big shopping mall called WANDA Plaza, where it is noisy and many people walk around and pass the line connected from the acoustic source and the user. The real experiment scenario is shown in Figure 6c. The sound pressure of the noisy shopping mall is highly unstable. It is between 55 and 75 dB SPL, which is shown in Figure 7b. We conduct four sets of experiments in total:

1. There is only one speaker broadcasting single high frequency acoustic signals of 20,500 Hz, *L* = 5 m, 10 m, 20 m, 30 m and $\beta =0^{\circ },\phi =45^{\circ }$ and $\theta =0^{\circ }$;
2. There is only one speaker broadcasting high frequency acoustic signals of 20,500 Hz and the common music, *L* = 5 m, 10 m, 20 m, 30 m and $\beta =0^{\circ },\phi =45^{\circ }$ and $\theta =0^{\circ }$;
3. There are two speakers in the environment. One of them is our target speaker. They broadcast different high frequency acoustic signals of 19,500 Hz and 21,500 Hz. To the target speaker, *L* = 5 m, 10 m, 20 m, 30 m and $\beta =0^{\circ },\phi =45^{\circ }$ and $\theta =0^{\circ }$;
4. There are two speakers in the environment. One of them is our target speaker. They broadcast different high frequency acoustic signals of 19,500 Hz and 21,500 Hz with the common music. To the target speaker, *L* = 5 m, 10 m, 20 m, 30 m and $\beta =0^{\circ },\phi =45^{\circ }$ and $\theta =0^{\circ }$.

We gather the mean angular errors of all the experiments. Then, we plot the CDF of the angular error in the noisy environment in Figure 12b. The results of experiments show that almost all errors are less than 12 degrees for L≤30M, which is acceptable for daily use.

### 5.5. System Efficiency

We evaluate the CPU usage when both the VULA and the VUCA system are running on the smartphone. The main cost for computation is the Band Pass Filter that we use. We find that when our system processes an acoustic wave, the usage of the CPU is 18% and the VUCA system is 22%. Both of these two systems’ processing times are within two seconds.

We also test battery consumption using both the VULA system and the VUCA system. During the non-stop experiment for one hour, the battery usage of our system is 15%. Therefore, we believe that normal usage of our systems will not significantly impact the battery life of a smartphone.

### 5.6. Nonuniform Circular Array

**Figure 13:**
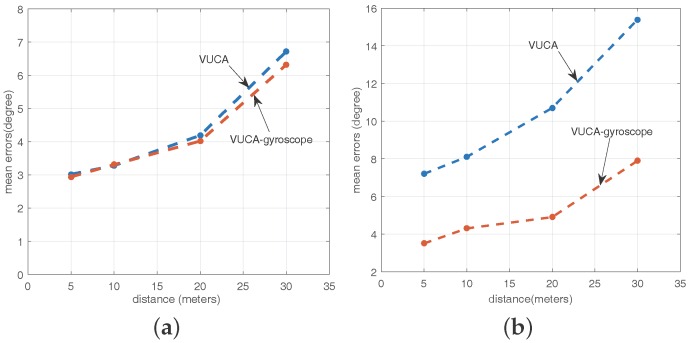
(**a**) mean errors of the VUCA system and the VUCA-gyroscope system with different distance and stable motion patterns; and (**b**) mean errors of the VUCA system and the VUCA-gyroscope system with different distance and unstable motion patterns.

According to the discussion in Section 4.8, we set some comparative experiments to verify the VUCA system’s robustness in a nonuniform circular array circumstance in the underground parking lot. First, we try to rotate our body naturally and stably during sampling, and we believe the angular velocity of our smartphone in these cases is approximately constant. Then, we detect the anchor points in the environment using both the regular VUCA system and the VUCA-gyroscope system. In Figure 13a, it shows the mean error of direction finding using two systems while *L* = 5 m, 10 m, 20 m, 30 m, $\beta =0^{\circ }$, $\phi =45^{\circ }$ and $\theta =0^{\circ }$.

Theoretically, the VUCA-gyroscope system should be better than the VUCA system by getting rid of the impact of the changing velocity of the smartphone. However, we find that there are not many differences between the performances of these two schemes in normal cases. We believe that adding the gyroscope to our system brings the increase in system calculation quantity. Thus, we need to reduce the number of sampling points to guarantee the response speed of our system. In addition, when we rotate our body, the smartphone itself may slightly spin in our hands. It will cause the output error of the gyroscope.

After that, we also try to rotate our body in more unstable patterns during the VUCA system and the VUCA-gyroscope system sampling progress. For example, we stop rotating during sampling for a few seconds. Then, we detect the anchor points in the environment using both the VUCA system and the VUCA-gyroscope system. We set the same experimental variables with Figure 13a. However, through the observation of Figure 13b, we find that the accuracy of the VUCA system has been seriously affected under this particular circumstance, while the VUCA-gyroscope performs much better.

**Figure 14:**
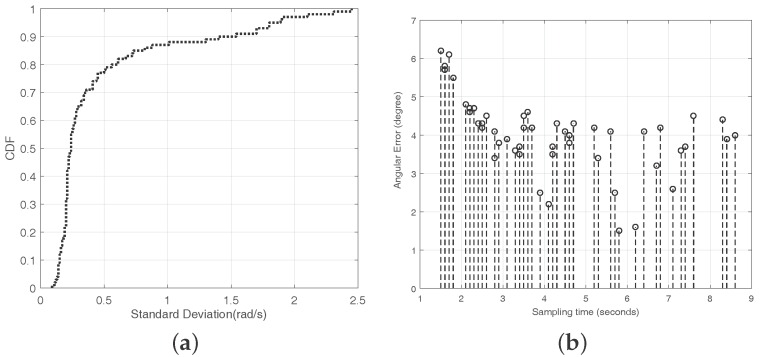
(**a**) the CDF of the standard deviation of the angular velocity; and (**b**) the angular errors of different sampling times.

Considering the efficiency and accuracy of the direction finding, we decide to utilize the VUCA-gyroscope system only when the standard deviation of the changing angular velocity during sampling is higher than the threshold we set. We let the experimenter try to rotate his body with the smartphone 50 times in natural patterns. After that, through the outputs of the gyroscope, we calculate the standard deviation of the shifty angular velocity during every sampling process. Finally, we plot the CDF of these results in Figure 14a. According to the extensive experiments, we decide to set the threshold value to 1 rad/s.

### 5.7. Different Sampling Time

We also analyze the angular errors caused by the different sampling time. We set *L* = 20 m, $\beta =\;\theta =0^{\circ }$ and $\phi =45^{\circ }$. A timer is installed in our VUCA system, and it records the sampling time of the whole direction finding progress. The experimenter rotates his body stably and naturally in different velocities for 50 times, and the timer records different sampling times. In the above experiments, the sampling time is between 2 and 4 s most of the time. However, we try to finish the sampling process between 1 and 9 s in this experiment, and then observe the impact of different sampling times on the system accuracy. According to Figure 14b, the results show that when the whole sampling time is less than 2 s, the accuracy of the VUCA system is affected. The reasons for this phenomenon are that when the sampling time is too short, the velocity of the body rotation is changed rapidly, and the experimenter may wobble his body or spin the smartphone in his hand. The sampled acoustic signals is not ideal under this circumstance.

### 5.8. Comparison with Swadloon and Borealis

To examine the optimality of our system, we compare our system with Swadloon [3] and Borealis [5] in Table 4. First, the direction finding accuracy of our system and Swadloon is acceptable in indoor environments. However, Borealis’s accuracy of direction finding is more suitable for outdoor scenarios. The received signal strength (RSS) measurements are not precise enough for indoor direction finding; Second, the Swadloon system and the VULA system have the Blind Zone problem. When the user turns his back on the acoustic source, the accuracies of these systems will be significantly affected. However, the VUCA and Borealis provide $360^{\circ }$ azimuth coverage. Third, due to the MUSIC algorithm we leverage, the VUCA system and VULA system have the features of anti-multipath. This feature will ensure the stability of our system in a heavy multi-path environment. Finally, the user’s motion patterns of using these systems all fall within the range of acceptable behaviors.

## 6. Conclusions

In this paper, we propose a novel scheme to estimate the direction of the high frequency acoustic source in indoor environments. By rotating a common smartphone around the user’s body for $360^{\circ }$, our system can imitate a VUCA to detect the direction of the user’s destination. Compared to other direction finding schemes, our solution does not require any specialized devices or fingerprints. In addition, it is accurate and efficient. In contrast to our previous work of the VULA system, we solve the Blind-zone problem and reduce the impact of elevation angle between the acoustic source and smartphone to increase the accuracy of direction finding. In addition, the nonuniform array problem has been well addressed by using the gyroscope of the smartphone. Eventually, by getting rid of the multipath effect using the MUSIC algorithm, the extensive experiments show that our scheme works well in various indoor environments. In addition, we can optimize the algorithm of the VUCA system in order to obtain faster response speed of our system in the future. In addition, we can try to develop an indoor localization system, which is based on our direction finding technique.

## Figures and Tables

**Table 1:** Standard deviation when user stands close to a wall.

Distance	2 m	8 m	15 m	20 m
**Standard Deviation**	$8.7^{\circ }$	$3.9^{\circ }$	$4.2^{\circ }$	$4.5^{\circ }$

**Table 2:** Standard deviation of non-line of sight circumstances.

Position of Blocker	1 m	3 m	5 m	7 m	9 m
**Standard Deviation**	$4.3^{\circ }$	$3.7^{\circ }$	$3.6^{\circ }$	$3.8^{\circ }$	$4.1^{\circ }$

**Table 3:** Different frequencies acoustic sources broadcast by different loudspeakers.

	Single Source	Double Sources	Triple Sources
**Mean Error**	$3.4^{\circ }$	$3.6^{\circ }$	$3.7^{\circ }$

**Table 4:** System comparison.

System	Direction Finding Accuracy	Blind Zone	Anti-Multipath	User’s Motion Pattern
VULA	$4.01^{\circ }$(Mean Error)	Yes	Yes	Shake or move the smartphone
VUCA	$3.4^{\circ }$(Mean Error)	No	Yes	$360^{\circ }$ body rotation
Swadloon	$2.1^{\circ }$(Mean Error)	Yes	No	Shake or move the smartphone
Borealis	80% less than $30^{\circ }$	No	No	$360^{\circ }$ body rotation
